# The Key Role of Government in Addressing the Pandemic of Micronutrient Deficiency Conditions in Southeast Asia

**DOI:** 10.3390/nu7042518

**Published:** 2015-04-08

**Authors:** Theodore H. Tulchinsky

**Affiliations:** 1 Braun School of Public Health and Community Medicine, Hebrew University-Hadassah, Hadassah Medical Center, Ein Karem, Jerusalem 91220, Israel; E-Mail: tulchinskyted@hotmail.com or tedt@hadassah.org.il; Tel.: +972-8-6781099; 2 School of Health Professions, Ashkelon Academic College, Ashkelon 78108, Israel

**Keywords:** micronutrient deficiency conditions, nutritional security, fortification of basic foods, governmental public health policy, private sector food processing, Millennium Development Goals

## Abstract

Micronutrient deficiency conditions are a major global public health problem. While the private sector has an important role in addressing this problem, the main responsibility lies with national governments, in cooperation with international agencies and donors. Mandatory fortification of basic foods provides a basic necessary intake for the majority and needs to be supported by provision of essential vitamin and mineral supplements for mothers and children and other high risk groups. Fortification by government mandate and regulation is essential with cooperation by private sector food manufacturers, and in the context of broader policies for poverty reduction, education and agricultural reform. Iron, iodine, vitamin A, vitamin B complex, folic acid, zinc, vitamin D and vitamin B12 are prime examples of international fortification experience achieved by proactive governmental nutrition policies. These are vital to achieve the Millennium Development Goals and their follow-up sustainable global health targets. National governmental policies for nutritional security and initiatives are essential to implement both food fortification and targeted supplementation policies to reduce the huge burden of micronutrient deficiency conditions in Southeast Asia and other parts of the world.

In this issue of *Nutrients*, an article by Gayer and Smith reports on a Workshop addressing micronutrient deficiency conditions in Southeast Asia and the role of the private sector in its alleviation and prevention [1]. This Commentary will address this pivotal issue of population nutritional security from a different perspective. The topic is vital to public health not only in the Southeast Asia region, but globally for all age groups, including in high income countries.

Micronutrient deficiency conditions of nutritional insecurity are widespread among an estimated 2 billion people in both developing and developed countries. These “silent epidemics” of vitamin and mineral deficiencies affect people of all genders and ages, as well as certain risk groups, most importantly for women, children and the elderly [2,3].

Micronutrient deficiency conditions not only cause specific diseases, but exacerbate complications of infections, such as measles, tuberculosis and diarrheal diseases, as well as chronic diseases, such as cancers and HIV/AIDS, greatly impacting on morbidity, mortality, and quality of life. Preventing such deficiencies in groups of people at special risk requires vitamin and mineral supplementation. But meeting community health needs for many micronutrient deficiencies safely and inexpensively is best met by population-based approaches involving fortification of commonly used basic foods.

Global nutritional health policies up to the 1980s for developing countries focused primarily on protein energy malnutrition, which still co-exists with widespread poverty. But there has been a gradual increase in recognition and increase in efforts to address the more prevalent and largely subclinical micronutrient deficiencies. Over recent decades, there have been greater awareness and increasing efforts to understand and control them, applying lessons learned in many high income countries which are still relevant to high income countries but most especially to low and medium income countries (LMICs).

In 1999, the United States Centers for Disease Control and Disease Prevention in Atlanta celebrated the great achievements of public health of the 20th century in a 10-part series in Morbidity and Mortality Weekly Reports (MMWR). Many of the deficiency diseases seen in the 19th and early 20th century were thought to be due to infectious diseases, but were shown to be due to dietary deficiency. The concept that “vital amines” could cause disease was first published in 1912, followed shortly thereafter by Goldberger’s investigation of a huge pellagra epidemic in the southern United States. Highlighted in this series was the control and near eradication of highly prevalent nutritional disorders that affect large percentages of the population in developed countries in the early 20th century and which are still widespread globally in the 21st century. The issue on safer and healthier foods [4] described these advances as follows:

“*The discovery of essential nutrients and their roles in disease prevention has been instrumental in almost eliminating nutritional deficiency diseases such as goiter, rickets, and pellagra in the United States. During 1922–1927, with the implementation of a statewide prevention program, the goiter rate in Michigan fell from 38.6% to 9.0 %. In 1921, rickets was considered the most common nutritional disease of children, affecting approximately 75% of infants in New York City. In the 1940s, the fortification of milk with vitamin D was a critical step in rickets control*.”

Many countries around the world suffer from vitamin and mineral deficiency conditions and are failing to meet the Millennium Development Goals (MDG) targets, especially those related to maternal and child health [5]. In 2010, Yach *et al.* address combating under-nutrition in developing countries through fortification of staple foods placing great emphasis on the role of the food industry in [6]. This stress on the private sector is mirrored in the workshop reported by Gayer and Smith [1].

Micronutrient deficiencies are addressed by complementary methods of fortification and supplementation, along with food security, education, and monitoring; all are challenges for public health clinical medicine and social policies. Micronutrient deficiencies in children relate mainly to vitamin A and D, iron and iodine deficiency, with clinical and sub-clinical rickets and anemia, as well as multiple deficiencies which harm child growth and development. Chronic diseases among adults, including osteoporosis, osteomalacia, thyroid deficiency, colorectal cancer and cardiovascular diseases are linked to deficiencies of iodine and vitamin D [7]. Such deficiencies increase the severity of infectious diseases such as measles, HIV/AIDS and tuberculosis.

Fortification has nearly a century long record of success and safety, proven effective for prevention of specific diseases, including birth defects. Understanding the pathophysiology and epidemiology of micronutrient deficiencies, and implementing successful methods of prevention, are both vital for nutritional security in contemporary public health standards, with examples of folic acid, vitamins A, B complex including B12, and vitamin D [8]. Nutrition policies need to be part of a broader context of multisectoral programming addressed societal determinants of under-nutrition, such as poverty reduction, gender equality, agricultural reform and health systems policies [9].

The Canadian Public Health Association reports that Canada has been a leading proponent of mandatory fortification of key basic foods to prevent micronutrient deficiencies. In the early 1900s, beriberi and blindness in segments of the population in Newfoundland and Labrador which led to the mandatory addition of calcium, iron, and B vitamins to flour and vitamin A to margarine in the 1940s. In 1946, nutrition surveys in two provinces estimated that about 21% of children had at least one sign of clinical vitamin A deficiency and that some 50% of school children had evidence of past rickets. Iodization of salt became mandatory in 1949, and eliminated goiter in Canada. During World War II, milk was fortified with vitamin D but the practice lapsed after the war, and rickets began to reappear. In 1965 regulations were enacted for mandatory addition of vitamin D to fluid milk which reduced the widespread problem with childhood rickets. Canada’s first comprehensive national nutrition survey conducted in 1970–1972 found many segments of the population had dietary intake inadequacies, especially iron, calcium, vitamin D, and protein. This led to mandatory fortification of salt with iodine, milk with vitamin D and flour with iron and vitamin B complex in 1979. In 2005, Health Canada reviewed and renewed mandatory food fortification regulations requiring the addition of Vitamin D to milk and folic acid to flour and to restore B vitamins and iron lost through food processing [10].

The US and Canadian populations are largely dependent on fortified foods and dietary supplements to meet basic nutritional needs of vitamin D, because foods naturally rich in vitamin D are limited and sun exposure varies by season, ethnicity, concern for skin cancer and changing life habits of children, young adults and older people. Fluid milk and breakfast cereals are the predominant vehicles for vitamin D in the United States, and Canada mandates fortification of fluid milk and margarine [11].

Since the 1980s, proof of the effectiveness of folic acid for prevention of some 70% of birth defects of the central nervous system (neural tube defects or NTDs), if taken in adequate amounts before pregnancy, created a new impetus for mandatory fortification policies. Attempts to have women in the age of fertility take folic acid pills at best reached one third of women, so that Canada, the United States, and Chile adopted mandatory fortification of flour in 1998 as the preferred method of reaching the entire population at risk and many countries have adopted mandatory fortification of flour with folic acid.

MMWR reported that from 2004 to 2007 the number of countries with national regulations for mandatory wheat-flour fortification increased from 33 to 54. All countries in North, Central and South America adopted mandatory fortification with folic acid so that the Americas Region of WHO the portion of wheat flour being fortified increased from 90% to 97%. In the African Region, fortified flour was increased from 26% to 31%; in South East Asia, from 16% to 21%; in the European Region from 3% to 6%; and in WHO’s Western Pacific Region from 2% to 4% [12].

Globally as of December 2014, 82 countries mandate fortification: 81 countries plus Punjab province in Pakistan have legislation to fortify wheat flour; of these 12 countries legislate fortification of maize products; and 6 countries mandate fortification of rice [13]. An example in Southeast Asia is the Policy—Philippine Food Fortification Act of 2000 which mandates fortification of rice with iron; wheat flour with vitamins A and Iron; refined sugar with vitamin A; cooking oil with vitamin A; and other staple foods with nutrients as required by the Governing Board of the National Nutrition Council [14].

In low- and medium-income countries both urban and rural populations are increasingly consuming processed food products such as milk and milk products, commercial baked goods, flour, oil, sugar, and salt. Fortification of basic processed foods has proven successful and well within the technical capacity of local industry in medium and low income countries. The enormous experience of fortification includes hundreds of millions of person years exposure over many decades with safe, inexpensive and highly effective fortification of salt with iodine; flour with iron and B-complex vitamins (including folic acid); and others, such as sugar with vitamin A and zinc (as done in Latin America), in keeping with the 2006 World Health Organization Guidelines for Fortification of Basic Foods [2].

Food fortification will not happen without strong and effective governmental public health policies and leadership but this must also be accompanied by policies and provision of selected or multi-vitamin supplements for at-risk groups such as women and children [15,16]. Public health professionals and policymakers in national, state and local government, as well as international health and food related agencies, have the moral responsibility to promote aggressive national nutrition policies [17]. Nigeria’s highly successful mandatory salt iodization program and vitamin A in maize, cooking oil and sugar and flour with iron, vitamins A, B complex demonstrate, with public and private sector, cooperation achievability of fortification policies in developing countries. Even developed countries in Europe need competent governmental leadership to adopt contemporary standards of food fortification and mandate fortification of flour with folic acid to prevent neural tube defects.

The issue of micronutrient deficiency conditions in Southeast Asia is no less severe than other parts of the LMIC world. Strong national and international leadership is needed to address this as is full cooperation and participation of the private sector. However, the key responsibility lies with government to establish policy and national objectives to set the rules and “level the playing field” in order to ensure that the essential nutrients reach all of the population and also that unregulated private fortification does not lead to exceeding the bounds of safety. Fortification, supplementation and monitoring are the trilogy of key public health issues for global nutrition security in our generation.
